# Porcine diazepam-binding inhibitor and bovine diazepam-binding inhibitor affect morphine antinociception via different receptors

**DOI:** 10.1007/s13238-016-0355-5

**Published:** 2016-12-28

**Authors:** Yu-Zhen Chen, Xiao-Cun Li, Zhen-Quan Guo, Li Zhou, Zhuan Zhou, Song-Ping Liang, Cai-Hong Wu

**Affiliations:** 1 State Key Laboratory of Membrane Biology, Peking University, Beijing, 100871 China; 2 College of Life Sciences, Peking University, Beijing, 100871 China; 3 PKU-IDG/McGovern Institute for Brain Research and Beijing Key Laboratory of Cardiometabolic Molecular Medicine, Institute of Molecular Medicine and Peking-Tsinghua Center for Life Sciences, Peking University, Beijing, 100871 China; 4 Institute of Clinical Medical Science, China-Japan Friendship Hospital, Beijing, 100029 China; 5 College of Life Sciences, Hunan Normal University, Changsha, 410006 Hunan China

**Dear Editor**,

Opioid addiction is one of the top challenges for society, particularly in China. To fight it, the key is to reveal its underlying mechanisms. Among the strategies to overcome the mental damage caused by opioids, investigating native anti-opioid peptides derived from mammalian (including human) brains is an important option because of safety concerns. In 1983, diazepam-binding inhibitor (DBI), a 10-kDa peptide, was first derived from rat brains (Guidotti et al., 1983). After repeated treatment with morphine, the DBI level is enhanced in rodent brains (Katsura et al., 1998; Shibasaki et al., 2006).

In the present work, we report an anti-opioid peptide-99A (AOP-99A) sample purified from porcine brain, the sequence of which is the same as that of the known DBI from pig intestine that reduces glucose-induced insulin secretion (Chen et al., 1988). We named it pDBI (Fig. 1A and 1B). Purified pDBI showed a single band on SDS-PAGE (Fig. 1C) and had a molecular weight of 9791 Da as determined by mass spectrometry (Fig. S1). Morphine antinociception was suppressed by purified pDBI (2.0 nmol/L, 4 μL i.c.v., Fig. 1D).

**Figure 1:**
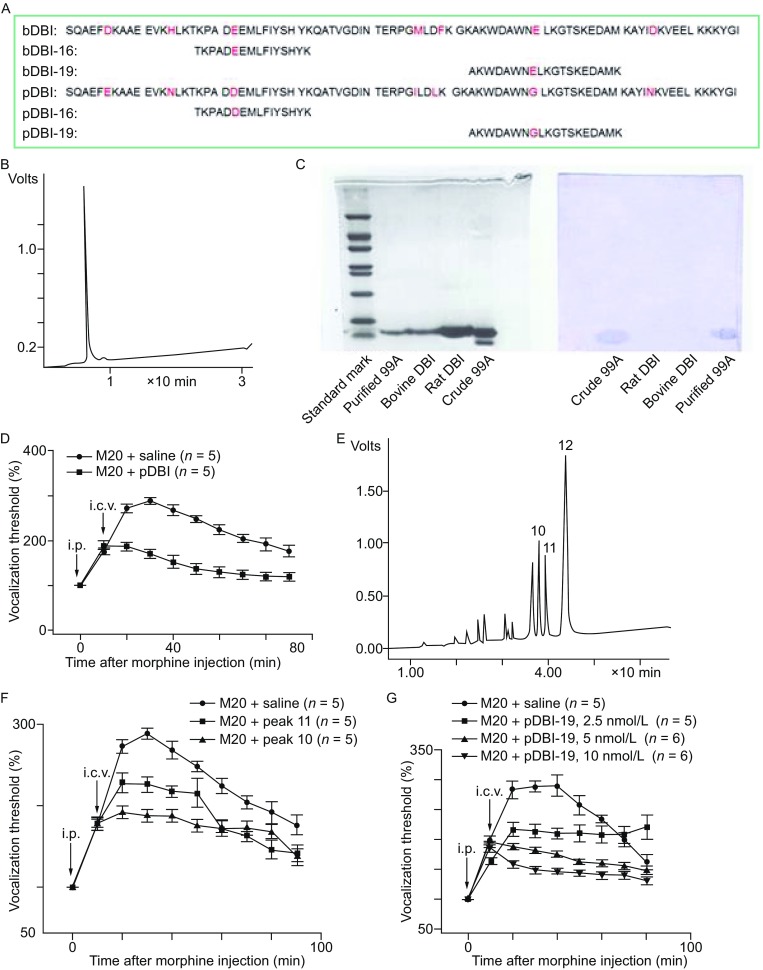
**Structures of porcine DBI, bovine DBI, and their active fragments**. (A) Schematic of the amino-acid sequences of pDBI and bDBI peptides and the four active fragments. (B) pDBI purified by HPLC on a C18-reverse-phase column. (C) pDBI showed a single band on SDS-PAGE (12%) and semi-dry western blotting. (D) Effect of pDBI (2 nmol/L, i.c.v.) on morphine antinociception (20 mg/kg, i.p.) at different time. Comparation of control/saline (*n* = 5) and pDBI groups (*n* = 5), *P* < 0.01, one-way ANOVA, followed by student’s t-test. (E) Hydrolysate of pDBI with trypsin separated by reversed-phase HPLC. (F) Bioactivity of samples from peaks 11 and 10. Comparation of saline, peak 10, and peak 11 groups (*n* = 5), *P* < 0.01. (G) Effect of pDBI-19 on morphine antinociception 10 min after morphine administration (20 mg/kg, i.p.). Comparation of saline, pDBI-19 (2.5 nmol/L), pDBI-19 (5.0 nmol/L), and pDBI-19 (10 nmol/L) groups (*n* = 6), *P* < 0.01

There is known to be 92% homology between the amino-acid residues of pDBI/AOP-99A and bovine DBI/endozepine (or bDBI, Figs. 1A, S2). The different amino residues of the two DBI-subfamilies are at positions 6, 14, 22, 46, 49, 60, and 75 from the N-terminal. Western blotting showed a positive immuno-response of pDBI to McAb-B2 against pDBI, but not bovine or rat DBI. This showed that pDBI differs from bDBI (Figs. 1C and S3). By degradation of pDBI with trypsin and separation by micro-HPLC, pDBI was separated into 12 peaks (Fig. 1E). Among these, the samples from peaks 10 and 11 substantially suppressed morphine antinociception (Fig. 1F).

In order to determine the sequences of the samples from peaks 10 and 11, two peptides pDBI-16 (99A-16) and pDBI-19 (99A-19) were synthesized based on residues 17–32 and 53–71 (Fig. 1A). Functional experiments showed that morphine antinociception was suppressed by i.c.v. injection of pDBI-16 (2.5, 5.0, and 10 nmol/L, Fig. S4) and pDBI-19 (2.5, 5.0, and 10 nmol/L, Fig. 1G). Thus, we found that the effect of pDBI-16 and pDBI-19 on morphine antinociception was same as that of full-length pDBI.

In order to reveal the bioactivity of bDBI-16 and bDBI-19, the fragments consistent with the 17–32 and 53–71 residues in bDBI were synthesized (Fig. 1A). Like full-length pDBI, pDBI-16, pDBI-19, and bDBI-16 (10, 20, and 40 nmol/L) also had a suppressive effect (Figs. 2A, C, and S3). However, the morphine antinociceptive effect was potentiated by bDBI-19 (Fig. 2B and 2D). This effect was similar to the potentiating effect of bDBI (i.c.v. in the 2–4 nmol/L dose range) on morphine antinociception (Chen et al., 1991).

**Figure 2:**
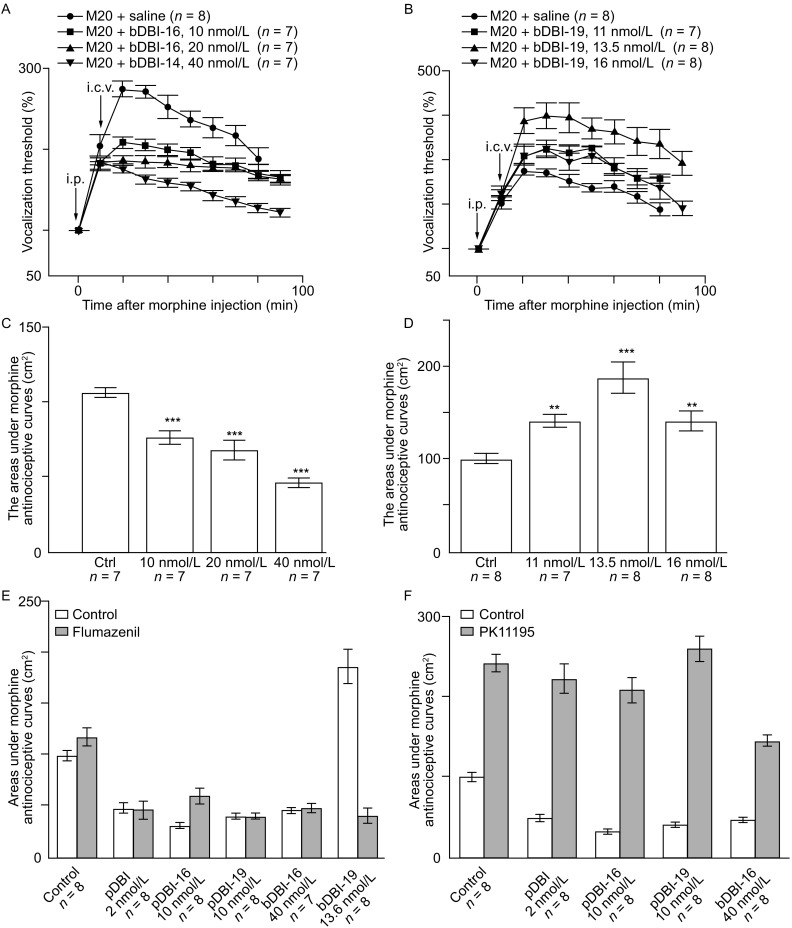
**Two functional subtypes of DBI peptides and morphine-antinociception: enhancer versus inhibitor**. (A) Morphine antinociception was significantly suppressed by bDBI-16 (i.c.v.). Comparation of saline, bDBI-16 (10 nmol/L), bDBI-16 (20 nmol/L), and bDBI-16 (40 nmol/L) groups (*n* = 7) 10 min after morphine injection (20 mg/kg, i.p.). (B) Potentiating effect of bDBI-19 on morphine antinociception. (C) Dose-response curve for suppressed morphine antinociception induced by bDBI-16 (**P <0.05, ***P <0.001 compared to control). (D) Dose-response curve for potentiated morphine antinociception induced by bDBI-19 (**P <0.05, ***P <0.001 compared to control). (E) In mice treated with flumazenil, the morphine antinociception induced by pDBI, pDBI-16, pDBI-19, and bDBI-16 was still suppressed, but the potentiated morphine antinociception induced by bDBI-19 was reversed (*n* = 8, *P* <0.001, student’s t-test). (F) In mice treated with PK11195, the suppressed morphine antinociception induced by pDBI, pDBI-16, pDBI-19, and bDBI-16 was fully removed

DBI is a 10-kDa polypeptide that was first isolated by monitoring its ability to displace diazepam from the benzodiazepine (BDZ) recognition site located on the extracellular domain of the GABA_A_ receptor and on the mitochondrial membrane (Guidotti et al., 1983; Costa and Guidotti, 1991). BDZ receptors are classified into two types: a central type linked to the GABA_A_ receptor-chloride channel complex, and a peripheral type not linked to the GABA_A_ receptor (Costa and Guidotti, 1991). The peripheral type is expressed at high levels on the outer mitochondrial membrane, particularly in astrocytes (Anholt et al., 1986; Costa and Guidotti, 1991). When mice were pretreated with flumazenil, an antagonist of the central type of BDZ receptor, the suppressed morphine antinociception was still induced by i.c.v. injection of saline, pDBI (2.0 nmol/L), pDBI-16 (10 nmol/L), pDBI-19 (10 nmol/L), and bDBI-16 (40 nmol/L). To our surprise, the potentiated morphine antinociception induced by bDBI-19 (13.6 nmol/L) was reversed in flumazenil-pretreated mice (Fig. 2E). However, in mice pretreated with PK-11195, an antagonist of the peripheral type of BDZ receptor, the suppressed morphine antinociception induced by i.c.v. injection of pDBI (2.0 nmol/L), pDBI-16 (10.0 nmol/L), pDBI-19 (10.0 nmol/L), and bDBI-16 (40 nmol/L) was lost (Fig. 2F). Our experiments, for the first time, revealed that the anti-morphine effect of pDBIs and bDBI-16 is mediated by the peripheral type of BDZ receptor, while the morphine-enhancing effect of bDBI-19 is mediated by the central type of BDZ receptor (Fig. 2E and 2F). There is a long lasting open question how i.v. bDBI can produce its enhancing-morphine effect same as by i.c.v. bDBI (Chen et al., 1991b), because bDBI (10 kDa) is too large to permeate the BBB, which permeates substance <3 kDa only. This puzzle is now solved, because intravenous bDBI is degraded in the serum into smaller peptides, including the blood-brain barrier-permeable bDBI-19 (2 kDa), which produces the bDBI-like enhancing-morphine effect (Fig. 2B and 2D).

We propose that the opposite effects of full-length pDBI and bDBI on morphine antinociception (Chen et al., 1991) are due to the opposing hydrophobicity of residues at position 60 and the different types of BDZ receptors. Interestingly, bDBI potentiates morphine effect of antinociception, but its 2 active fragments bDBI-16 and bDBI-19 show opposite effects (Figs. 2 and S4). The underlying mechanisms are unclear. Future work is needed to address the remaining questions (1) whether the peripheral type of BDZ receptor is sensitive to bDBI-19, and (2) why bDBI-16 has an effect opposite to bDBI and bDBI-19.

It is known that the amino-acid DBI-E60 is conserved in rat, mouse, cow, and human (Costa and Guidotti, 1991) but not in porcine DBI derived from pig intestine. The function of pDBI and its fragments (pDBI-33–50 and pDBI 17–50) are associated with various neuropsychiatric disorders involving neuronal excitability (Costa and Guidotti, 1991). In the present work, we discovered that pDBI has two active fragments (pDBI-16 and pDBI-19) that suppress morphine antinociception. Thus, pDBIs might play important roles during morphine tolerance and dependence (data not shown). It is known that DBI concentration increases in mammals following continuous treatment with morphine (Katsura et al., 1998; Shibasaki et al., 2006). Therefore, we suggest that pDBI and bDBI and the active fragments may co-exist in the central nervous system and play multiple physiological roles during the development of morphine tolerance and dependence. Although it remains unclear how pDBIs and bDBIs are produced after morphine intake in humans, our study provides a new mechanism of morphine tolerance and dependence underlying addiction in animals which may help future efforts treating addicted patients.

## Electronic supplementary material

Below is the link to the electronic supplementary material.

Supplementary material 1 (PDF 500 kb)

## References

[CR1] Anholt RR, Pedersen PL, De Souza EB, Snyder SH (1986). The peripheral-type benzodiazepine receptor. Localization to the mitochondrial outer membrane. J Biol Chem.

[CR2] Chen ZW, Agerberth B, Gell K, Andersson M, Mutt V, Ostenson CG, Efendic S, Barros-Soderling J, Persson B, Jornvall H (1988). Isolation and characterization of porcine diazepam-binding inhibitor, a polypeptide not only of cerebral occurrence but also common in intestinal tissues and with effects on regulation of insulin release. Eur J Biochem.

[CR3] Chen YZ, Wang JY, Zhou S, Shoyab M (1991). Bovine endozepine potentiates morphine analgesia in mice. Life Sci.

[CR4] Costa E, Guidotti A (1991). Diazepam binding inhibitor (DBI): a peptide with multiple biological actions. Life Sci.

[CR5] Guidotti A, Forchetti CM, Corda MG, Konkel D, Bennett CD, Costa E (1983). Isolation, characterization, and purification to homogeneity of an endogenous polypeptide with agonistic action on benzodiazepine receptors. Proc Natl Acad Sci USA.

[CR6] Katsura M, Hara A, Higo A, Tarumi C, Hibino Y, Ohkuma S (1998). Continuous treatment with morphine increases diazepam binding inhibitor mRNA in mouse brain. J Neurochem.

[CR7] Shibasaki M, Katsura M, Tsujimura A, Ohkuma S (2006). Up-regulated L-type high voltage-gated calcium channels cause increase in diazepam binding inhibitor induced by sustained morphine exposure in mouse cerebrocortical neurons. Life Sci.

