# Supplementary material for: Porcine diazepam-binding inhibitor and bovine diazepam-binding inhibitor affect morphine antinociception via different receptors
Source: Protein Cell. 2016 Dec 28;8(2):140–3. doi: 10.1007/s13238-016-0355-5 (PMC5291778; doi:10.1007/s13238-016-0355-5)
Supplement: Supplementary file 1 — Supplementary material 1 (PDF 500 kb) [file 13238_2016_355_MOESM1_ESM.pdf]

SUPPLEMENTAL MATERIALS

Protein production

The gene of full-length hDDX41 protein (GenBank No. NP_057306.2) was provided by professor Yong-Jun Liu and sub-cloned into pMCSG7 vector using a ligation-independent cloning method (Stols et al., 2002). The construct was transformed into *Escherichia coli* (*E.coli*) BL21 (DE3) cells. The cells were cultured in LB medium containing 100 μg/ml ampicillin at 37°C until the $OD600_{nm}$ reached 0.8, and then induced with 0.2 mM isopropylthio-beta-D-galactoside (IPTG) for 20 h at 16°C. Cells were harvested by centrifugation (4,000 rpm, 30 min) and re-suspended in PBS buffer (137 mM NaCl, 2.7 mM KCl, 50 mM Na_2HPO_4, 10 mM KH_2PO_4, pH 7.4). After ultrasonication and centrifugation (18,000 rpm, 30 min), the supernatant was loaded onto a Ni-NTA column (Qiagen) equilibrated with PBS buffer. The column was firstly washed with 20 ml PBS, followed by washing with 50 ml PBS containing 20 mM and 50 mM imidazole, and finally eluted with PBS containing 300 mM imidazole. The purified protein was examined by SDS-PAGE, and the N-terminal 6 × His-tag was removed by tobacco etch virus (TEV) protease. Uncut protein was separated by a second Ni-affinity chromatography step. Fractions containing the protein were concentrated and loaded onto Superdex S200 column (GE Healthcare) equilibrated with 20 mM Tris-HCl (pH 8.0), 150 mM NaCl, 10 mM $MgCl_2$. Fractions containing target protein were concentrated to 20 mg/ml and stored at -80°C for further use.

The expression level of full length DDX41 in BL21 (DE3) is very low. The gene was

then cloned into pFastBac HT A vector and expressed in Sf9 cells. Unfortunately, full length protein was unstable and degraded badly. At the same time, different truncations containing only DEAD domain or DEAD domain together with Helicase domain were constructed into pMCSG7 vector. All proteins were purified as described above. Two truncations containing amino acids 153-410 (DEAD domain) and 153-569 (DEAD domain and Helicase domain) were very stable, and proteins were screened for crystallization.

Crystallization and data collection

The purified amino acid (aa) 153-410 and 153-569 truncation proteins were screened for initial crystal screening by Mosquito Robot (TTP LabTech). No crystals grew up for aa 153-569. Crystals for aa 153-410 grew up in many conditions. Crystals were optimized by mixing 1 µl protein with 1 µl reservoir solution by hanging drop vapor diffusion methods at 16°C. The best diffracting crystals were obtained in the condition containing 0.2 M ammonium sulfate, 25% PEG 3,350, 0.1 M Bis-Tris, pH 6.5. Crystals were harvested and cryo-protected in the well solution containing an additional 25% (v/v) glycerol as a cryoprotectant and then flash cooled in a dry nitrogen stream. Diffraction data for aa 153-410 (DEAD domain) was collected at 100 K using an ADSC Q315 CCD detector on beamline BL17U1 of Shanghai Synchrotron Radiation Facility (SSRF).

Structure determination and refinement

The structure was solved by molecular replacement method with Phaser (McCoy, 2007) using the DEAD domain of DDX5 (PDB code: 3FE2) as the searching model. Model building and structure refinement were performed automatically in PHENIX (Adams et al., 2010) and manually in Coot (Emsley and Cowtan, 2004). Statistics for data collection and refinement are presented in Table 1.

Isothermal Titration Calorimetry (ITC)

ITC measurement was performed on an iTC200 calorimeter (Microcal Inc., Northampton, MA). The experiment was carried out at 20 °C in 20 mM Tris-HCl, pH 8.0, 150 mM NaCl, 10 mM $MgCl_2$. The reactant (50 μM aa 153-410 DEAD domain protein or 50 μM hDDX41 full length protein) was placed in the 300 μl sample chamber and ATP/ADP/AMP/c-di-GMP/cGAMP (500 μM) was added using the syringe with 20 successive additions of 2 μl for 4 s (with an initial injection of 0.5 μl). The interval between each injection lasted 120 s. The results were processed with the ORIGIN7 software (Microcal Inc).

Thermal Shift Assay (TSA)

Thermal shift assay was conducted using 2 μg protein with or without ATP/ADP/AMP/c-di-GMP/cGAMP and a 1000 dilution of SYPRO Orange dye (Invitrogen) in 20 mM Tris-HCl, pH 8.0, 150 mM NaCl, 10 mM $MgCl_2$. The fluorescence signals as a function of temperature were recorded using a Real-time PCR machine (Bio-Rad CFX96) in the FRET mode. The temperature gradient was

set in the range of 20-95°C with an increasement of 0.5°C over the course of 15 s.

Multiangle static light scattering

For assessment of the oligomeric state and molecular weight of full length and different truncations of hDDX41, a WTC-030S5 size exclusion column (Wyatt Technologies), equilibrated with 20 mM Tris-HCl, pH 8.0, 150 mM NaCl, 10 mM $MgCl_2$ was connected to a miniDAWN Tristar light scattering instrument (Wyatt Technologies). Data analysis was performed using the manufacturer's software ASTRA.

Cell culture, transfection and Intracellular localization of hDDX41

HEK293T cells were grown at 37 °C with 5% CO2 in DEME (Gibco) medium containing 10% fetal bovine serum. Transfection of DNA (pCI-neo-hDDX41-1-622-GFP/pCI-neo-hDDX41-153-622-GFP) into cells was performed using Lipofectamine 2000 (Invitrogen). Transfected cells were fixed in PBS containing 4% paraformaldehyde for 15 min, washed twice, and permeabilized in 0.1% Triton X-100 in PBS for 5–15 min. Nucleus was stained with DAPI. Localization of GFP-tagged proteins was analyzed by fluorescence microscope.

Accession code Atomic coordinate and structure factors have been deposited in Protein Data Bank with accession code 5H1Y.

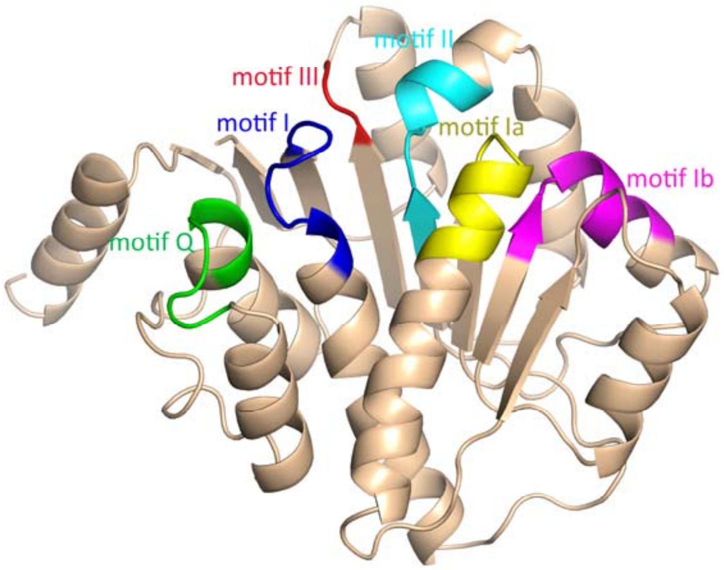

Figure S1. Conserved motifs of hDDX41 DEAD domain.

Conserved motifs of hDDX41 DEAD domain are located at one side of the structure, labeled by different colors.

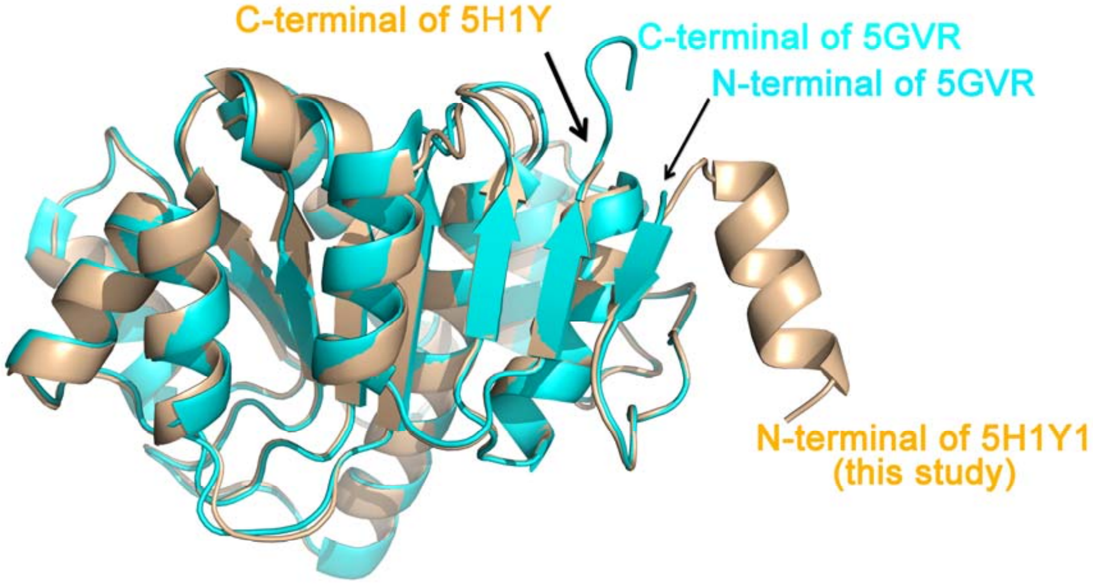

Figure S2. Structural comparison of our hDDX41 DEAD domain with apo form of DDX41 DEAD domain reported by Omura et al. (Omura et al., 2016).

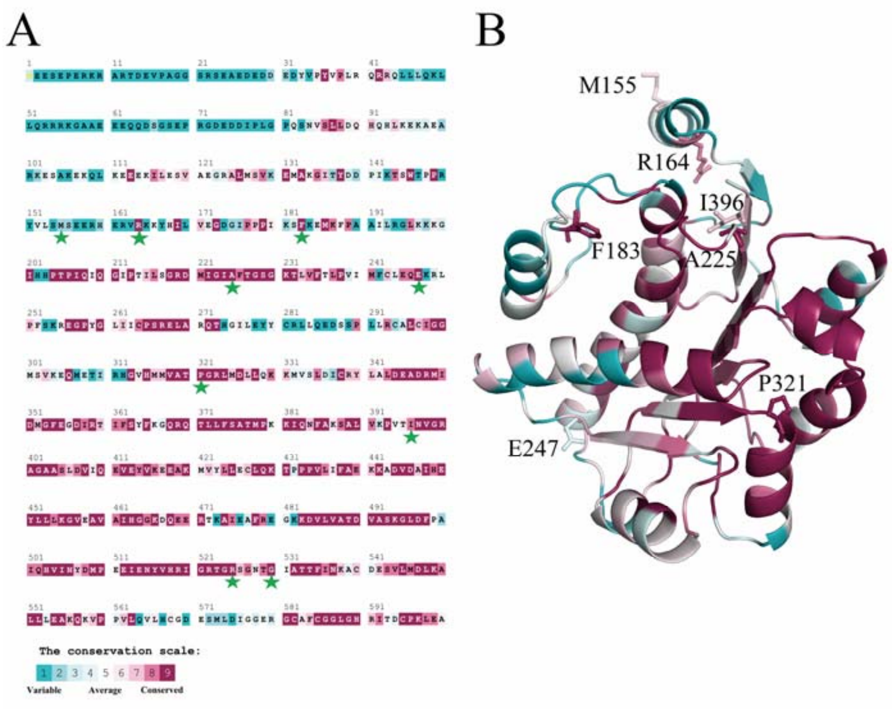

Figure S3. The conservation of amino acids and DEAD domain structure of hDDX41.

(A) Representation of amino acids (151-600) conservation by ConSurf server (http://consurf.tau.ac.il/). Color scale is based on level of conservation determined by the server. The mutations associated with MDS/AML syndrome are marked by green stars. (B) The conservation of DEAD domain structure. Mutations in DEAD domain are shown in sticks.

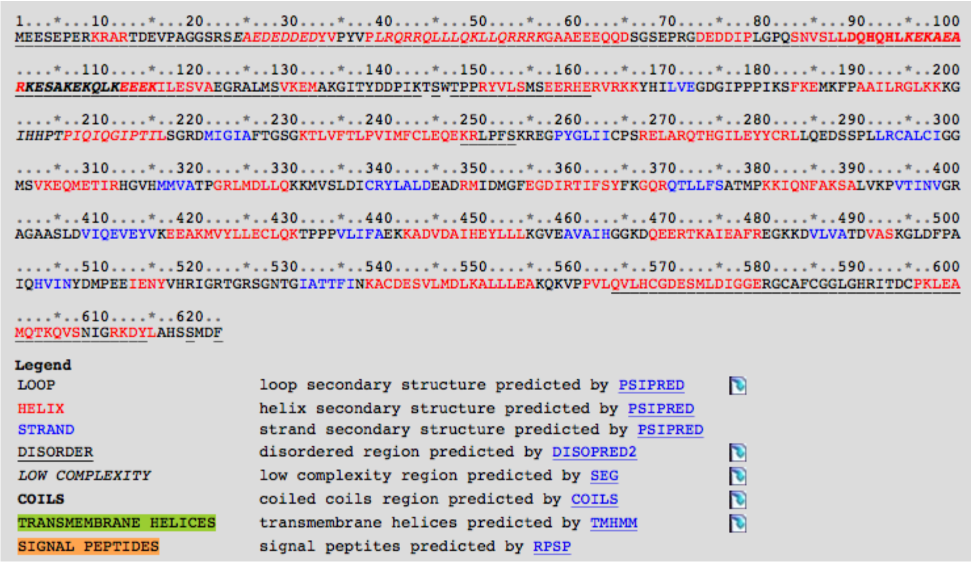

Figure S4. Secondary structure prediction of hDDX41.

Secondary structure prediction of hDDX41 by XtalPred-RF server (http://ff-as.burnham.org/XtalPred-cgi/xtal.pl) The N-terminal region (aa 1-160) is predicted to be disordered.

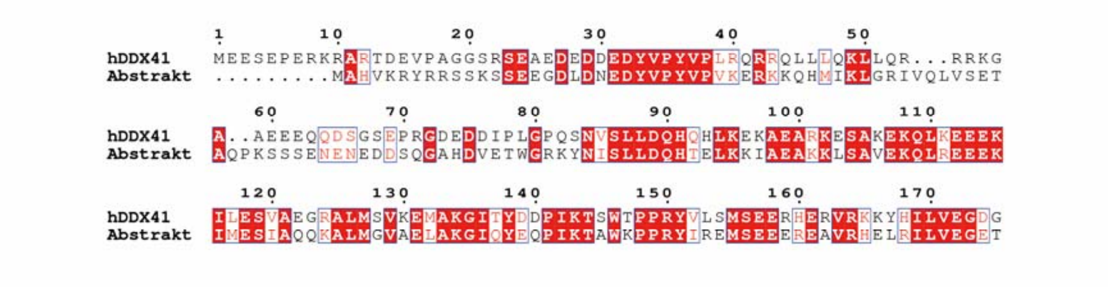

Figure S5. Sequence alignment of hDDX41 N-terminal region and its homologues Abstrakt.

Table S1. Data Collection and Refinement Statistics

Crystal	hDDX41(aa153-410)
X-ray source	SSRF/BL17U1
Crystal to detector distance (mm)	320
Number of images	180
Oscillation width (º)	1.0
Wavelength(Å)	0.98
Space group	$P2_1$
a, b, c (Å)	43.89, 62.75, 92.89
α, β, γ (°)	90.00, 94.32, 90.00
No.Protein Molecules/ASU	2
Resolution range(Å)	50.00-2.26
	(2.33-2.26)
Rsym (%)	6.9 (54.7)
Mean I/σ(I)	21.40 (3.34)
Completeness (%)	98.6 (97.0)
Redundancy	3.4 (3.4)
Refinement	
Resolution (Å)	35.89-2.26
No. reflections	23367
R_{work}/ R_{free} (%)	18.57/23.28
No. atoms	4028
No. Protein atoms	3870
No. Water	158
Mean B ($Å^2$)	32.24
R.m.s deviations	
Bond lengths (Å)	0.009
Bond angles (º)	1.234

Ramachandran analysis	
Favored region (%)	98.96
Allowed region (%)	1.04
Outliers (%)	0.00

Numbers in parentheses represent statistics for the highest resolution shell.

$R_{work} = \sum||Fobs| - |Fcalc||/ \sum|Fobs|$.

R_{free} = R factor for a selected subset (5%) of the reflections that were not included in prior refinement calculations.

Table S2. Interactions Between Motifs

Interactions between motif Q and motif I		
motif Q (residue [atom])	Dist. [Å]	motif I (residue [atom])
T205 [OG1]	2.9	S229 [O]
T207 [CG2]	3.3	S229 [O]
Q208 [NE2]	2.8	G230 [O]
Interactions between motif I and motif II		
motif I (residue [atom])	Dist. [Å]	motif II (residue [atom])
K231 [NZ]	2.7	D344 [OD1]
T227 [OG1]	3.8	E345 [OE2]
Interactions between motif I and motif III		
motif I (residue [atom])	Dist. [Å]	motif III (residue [atom])
A225 [O]	3.4	A376 [C]
A225 [N]	3.4	S375 [O]
Interactions between motif II and motif III		
motif II (residue [atom])	Dist. [Å]	motif III (residue [atom])
D347 [OD2]	3.2	S375 [OG]
D347 [OD2]	3.1	T377 [OG1]
Interactions between motif II and motif Ia		
motif II (residue [atom])	Dist. [Å]	motif Ia (residue [atom])
D344 [OD2]	3.6	L269 [CD2]
E345 [OE1]	3.1	P265 [CG]
Interactions between motif Ia and motif Ib		
motif Ia (residue [atom])	Dist. [Å]	motif Ib (residue [atom])
R267 [NH1]	2.8	R323 [NH2]

SI references

Adams, P.D., Afonine, P.V., Bunkoczi, G., Chen, V.B., Davis, I.W., Echols, N., Headd, J.J., Hung, L.W., Kapral, G.J., Grosse-Kunstleve, R.W., *et al.* (2010). PHENIX: a comprehensive Python-based system for macromolecular structure solution. Acta Crystallogr D Biol Crystallogr 66, 213-221.

Emsley, P., and Cowtan, K. (2004). Coot: model-building tools for molecular graphics. Acta Crystallogr D Biol Crystallogr 60, 2126-2132.

McCoy, A.J. (2007). Solving structures of protein complexes by molecular replacement with Phaser. Acta Crystallogr D Biol Crystallogr 63, 32-41.

Omura, H., Oikawa, D., Nakane, T., Kato, M., Ishii, R., Ishitani, R., Tokunaga, F., and Nureki, O. (2016). Structural and Functional Analysis of DDX41: a bispecific immune receptor for DNA and cyclic dinucleotide. Sci Rep 6, 34756.

Stols, L., Gu, M., Dieckman, L., Raffen, R., Collart, F.R., and Donnelly, M.I. (2002). A new vector for high-throughput, ligation-independent cloning encoding a tobacco etch virus protease cleavage site. Protein Expr Purif 25, 8-15.
